# The Use of Quantitative Sensation Testing to Identify the Physiological Differences Between the Median and Ulnar Nerves

**DOI:** 10.3389/fnhum.2021.601322

**Published:** 2021-05-06

**Authors:** Magdalena Koszewicz, Mariusz Szydlo, Jerzy Gosk, Malgorzata Wieczorek, Krzysztof Slotwinski, Slawomir Budrewicz

**Affiliations:** ^1^Department of Neurology, Wrocław Medical University, Wrocław, Poland; ^2^Department of Trauma and Hand Surgery, Wrocław Medical University, Wrocław, Poland; ^3^Faculty of Earth Sciences and Environmental Management, University of Wrocław, Wrocław, Poland

**Keywords:** median nerve, ulnar nerve, physiology of nerve conduction, sensory nerve conduction study, quantitative sensation testing

## Abstract

**Introduction:**

Similarities in morphology, physiological function, and neurophysiological findings between median and ulnar nerves are not unequivocal. Our previous study confirmed differences in motor fiber parameters between these nerves in healthy persons. We made an attempt to assess and compare the physiological parameters of different sensation modalities (temperature, pain, and vibration) in median and ulnar nerves.

**Methods:**

The study was performed in 31 healthy, right-handed volunteers: 17 women, 14 men, mean age 44.8 ± 15.5 years. Standard sensory conduction tests in the median and ulnar nerves were performed together with the estimation of vibratory, temperature, and warm- and cold-induced pain thresholds in the C7 and C8 dermatomes on the palm, using quantitative sensory testing.

**Results:**

There were no statistically significant differences in the standard sensory conduction test in the median and ulnar nerves across the whole group: between right and left hands, and between women and men. We revealed differences in the temperature and pain thresholds between these nerves, mainly in low temperature perception. There were no differences in estimated thresholds between sides or in female and male groups. The vibratory limits did not differ significantly between nerves, and subgroups.

**Conclusion:**

The study confirmed the differences in the physiological sensory perception between the median and ulnar nerves. The median nerve is more sensitive to temperature stimulation than the ulnar nerve, but simultaneously less sensitive to pain-inducing temperature stimuli. These findings should be considered during the examination of hand nerve pathology.

## Introduction

Human hand innervation depends on median and ulnar nerves. In the literature, there are discrepancies concerning similarities or differences in morphology between these nerves, e.g., as seen in ultrasound and morphometric analysis, different physiological function, and neurophysiological findings. This is important, because these nerves are often compared in the evaluation of hand nerve pathology, above all in carpal tunnel syndrome examination (Sable, 1998; Sander et al., 1999; Nouraei et al., 2015; Barton et al., 2016; Wyner and Dissabandara, 2018). In our previous study (Koszewicz et al., 2019) we showed faster motor conduction velocities in the ulnar nerve using a more advanced neurophysiological technique: the conduction velocity distribution (CVD) test. The findings confirmed the presence of more larger fibers in the ulnar than in the median nerve. Functional movement tasks of the hand could explain the differences found in the study. Melchior et al. (2007) found a significant correlation between sensibility test results (sensory light touch) and functional hand ability. Some observations presented in the literature highlighted the differences in the standard sensory conduction between median and ulnar nerves (Cruz Martínez et al., 1978; Alemdar, 2014). Quantitative sensation testing (QST) allows evaluation of thermal and mechanical perception throughout the determination of cold, warm and temperature-induced pain or vibratory thresholds. QST is a valuable instrument for the assessment of small fiber (A-delta and C fibers) function, which is impossible using standard electroneurographic tests (Siao and Cros, 2003; Nothnagel et al., 2017). The evaluation of differences in small fiber function between the median and ulnar nerves could be an additional procedure helpful in the reliable diagnostics of entrapment syndromes.

In our study, we decided to compare the sensory function in median and ulnar nerves in middle-aged, healthy persons. We based our investigations on the standard sensory conduction tests together with temperature, pain, and vibratory threshold estimations obtained in QST.

## Materials and Methods

The Ethics Committee of Wrocław Medical University in Poland gave its approval for the study. We obtained informed consent for participation in the study from all volunteers.

We examined the previously evaluated volunteer group (Koszewicz et al., 2019). 31 healthy, only right-handed volunteers (62 hands) with a mean age of 44.8 ± 15.5 years participated in the study. They fulfilled the same exclusion criteria as previously, i.e., absence of all diseases and conditions influencing the peripheral nervous system, and among others: all types of polyneuropathies, myopathies, hormonal, rheumatic, and malignant diseases, vitamin deficiency, exposure to toxins, and addiction to alcohol and drugs. Neurological examinations were performed in all persons in order to exclude clinical symptoms of polyneuropathy.

In the study, we used a Viking Quest version 10.0 device which was connected to a Thermal Sensory Analyzer II 2001 (TSA II) together with a VSA–3000 Vibratory Sensory Analyzer (Medoc, Israel).

Sensory conduction tests were performed according to standard procedures (Oh, 2003; Mallik and Weir, 2005). We used an antidromic technique, using ring recording electrodes and electrical stimuli at the wrist, at the second distal-most cease. The responses were obtained from the second digit for the median nerve, and from the fifth digit for the ulnar nerve. The distances between stimulating and recording electrodes varied from 11 to 13 cm for both nerves. Standard conditions were maintained: room temperature was 21–23°C, hand temperature was equal to or above 32°C. We estimated distal latency (in milliseconds–ms), amplitude (in microvolts–μV), and conduction velocity (in meters per second–m/s).

**FIGURE 1:**
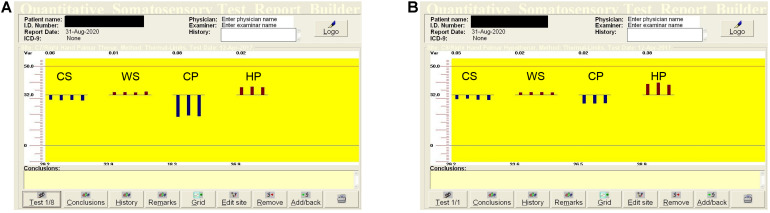
Quantitative sensory testing (QST) in the median **(A)** and ulnar **(B)** nerves in the right hand in a healthy, 51-years old woman. The dispersions of the temperatures are: for low temperatures (CP–CS) in the median nerve–10.9°C, in the ulnar nerve–2.7°C, for high temperatures (HP–WS) in the median nerve–3.0°C, in the ulnar nerve–5.3°C. QST, quantitative sensation testing; CS, cold sensation; WS, warm sensation; CP, cold pain; HP, heat pain.

Quantitative sensation testing allows estimation of the sensation and pain thresholds for cold and warm temperatures, and additionally uses a special device module–vibration threshold. The following thresholds were estimated using limit methods: cold sensation (CS), warm sensation (WS), cold pain (CP), heat pain (HP), and vibration sensation (VS). We also analyzed the temperature differences between CS and CP, and WS and HP (the dispersion of the temperature). Thermal stimuli were produced by a thermode (Peltier modules). The thermode active area is 30 × 30 mm, temperature range 0–50.5°C. The thermode was attached to the skin of the palm on the region of the C7 dermatome for the median nerve (thenar region), and the C8 dermatome (hypothenar region) for the ulnar nerve. The temperature changed by 1°C/s during temperature threshold estimation, and 2°C/s when the pain threshold was evaluated. The basic temperature (adaptation temperature) was 32°C. When the patients felt cold, warm or pain, the stimulation was stopped by pressing a button (subjective part of the study). The procedures were repeated four times for temperature threshold estimation, three times for pain. The thresholds were calculated as the average values in degrees Celsius (Shy et al., 2003; Siao and Cros, 2003; Rolke et al., 2006) and are shown graphically (Figure 1).

A vibratory sensation analyzer measures thresholds for vibratory stimuli. The vibration threshold represents the amplitude of vibration, and is calculated in microns (μ). Patients put their index finger (the median nerve) or small finger (the ulnar nerve) on a vibrating button with a stimulating area equal to 1.22 cm^2^. The vibratory stimulation rate was 100 Hz, the amplitude ranged from 0 to 130 microns (μ), the amplitude changed with a rate of 0.3 microns per second (μ/s). When the patients felt a vibration, the stimulation was stopped by pressing a button. The sensation threshold of vibration was calculated as an average value from 6 repetitions (Shy et al., 2003; Siao and Cros, 2003).

The statistical analysis was performed with STATISTICA 12.0 software (Statsoft Polska Sp. z.o.o, Krakow, Poland). The statistical analysis included: the number of cases (N), mean values (X) with standard deviations (SD) of the continuous parameters, the Shapiro-Wilk normality test and the student *t*-test for data with a normal distribution and homogeneity of variance, Mann–Whitney *U* test and Spearman test for data without a normal distribution. The significant *P*-value was ≤ 0.05. We used Guilford’s interpretation of the magnitude of significant correlations.

## Results

The study was conducted on a group of 17 healthy women, and 14 healthy men (31 volunteers, 62 hands). All participants were right-handed. Mean age was 44.8 ± 15.5 years: male group–42.8 ± 14.1, female group–46.7 ± 16.9 years.

All statistical analyses were performed for the study group, for right and left hands, and male and female groups. Standard sensory conduction parameters for the median and ulnar nerves are shown in Table 1. There were no statistically significant differences between amplitude and sensory conduction velocity in the median and ulnar nerves in the whole group, or in right and left hands, and in male and female groups. A difference was only seen between distal latencies in the median and ulnar nerves in the whole group (Table 1), in right (median–2.43 ± 0.61 ms, ulnar–2.08 ± 0.26 ms, *p* < 0.0003), and left hands (median–2.47 ± 0.50 ms, ulnar–2.12 ± 0.32 ms, *p* < 0.00008), but not between sides. A difference of distal latencies was also noted in men (median–2.26 ± 0.53 ms, ulnar–2.26 ± 0.34 ms, *p* < 0.0003) and women (median–2.30 ± 0.52 ms, ulnar–1.98 ± 0.17 ms, *p* < 0.00001), but not between gender groups. However, the latency parameter was not well standardized, because the distance between stimulating and recording electrodes varied from 11 to 13 cm in both nerves.

**TABLE 1:** Mean standard sensory conduction parameters in the median and ulnar nerves.

**Nerves**	**Median**			**Ulnar**			***p*-value**
	***n***	**mean**	**STD**	***n***	**mean**	**STD**	
Latency (ms)	62	2.45	0.55	62	2.10	0.29	<0.00001*
Amplitude (μV)	62	37.80	18.34	62	36.28	17.91	0.592
Conduction Velocity (m/s)	62	56.50	8.09	62	54.68	5.61	0.139

*The significant *P*-value ≤ 0.05.

Vibratory limits did not differ significantly between groups, there were no differences between nerves, sides or genders. The statistical significant differences between the median and ulnar nerves were seen in CS, WS, and CP thresholds for the whole group and subgroups. HP thresholds did not achieve statistical significance in any of the groups (Tables 2–4). CS was higher, and WS and CP were lower in the median nerve. The analysis of the temperature difference between pain and temperature sensations (cold and warm), i.e., the dispersion of the temperature feelings, showed the significant differences between median and ulnar nerves in the low temperature range: for median nerve–10.68°C, for ulnar nerve–7.58°C. The dispersion in the range of high temperatures in both nerves was similar (Table 5).

**TABLE 2:** Mean quantitative sensory testing (QST) parameters in the median and ulnar nerves.

**Nerves**	**Median**			**Ulnar**			***p*-value**
	***n***	**mean**	**STD**	***n***	**mean**	**STD**	
cold sensation (°C)	61	29.84	1.24	61	29.29	1.37	0.011*
warm sensation (°C)	61	33.98	0.76	61	34.43	1.31	0.030*
cold pain (°C)	60	19.26	5.90	60	21.60	5.62	0.017*
hot pain (°C)	61	40.35	3.64	61	40.57	4.06	0.935
vibratory limits (μ)	61	1.24	0.95	61	1.31	1.34	0.357

*The significant *P*-value ≤ 0.05.

**TABLE 3:** Mean QST parameters in the median and ulnar nerves on the left and right sides.

	**Left hand**						

**Nerves**	**Median**			**Ulnar**			***p*-value**
	***ni***	**mean**	**STD**	***ni***	**mean**	**STD**	
cold sensation (°C)	32	29.72	1.47	32	29.41	1.30	0.183
warm sensation (°C)	32	34.08	0.87	32	34.30	1.04	0.400
cold pain (°C)	32	19.02	5.78	32	21.66	5.73	0.028*
hot pain (°C)	32	40.13	3.25	32	40.11	3.62	0.920
vibratory limits (μ)	32	1.23	0.96	32	1.35	1.28	0.716

	**Right hand**						

**Nerves**	**Median**			**Ulnar**			***p*-value**
	***ni***	**mean**	**STD**	***ni***	**mean**	**STD**	

cold sensation (°C)	29	29.96	0.93	29	29.16	1.45	0.030*
warm sensation (°C)	29	33.86	0.59	29	34.58	1.57	0.021*
cold pain (°C)	28	19.54	6.12	28	21.53	5.61	0.225
hot pain (°C)	29	40.60	4.08	29	41.08	4.50	0.762
vibratory limits (μ)	29	1.24	0.96	29	1.27	1.43	0.385

*The significant *P*-value ≤ 0.05.

**TABLE 4:** Mean QST parameters in the median and ulnar nerves in the male and female groups.

	**Male**						

**Nerves**	**Median**			**Ulnar**			***p*-value**
	***ni***	**mean**	**STD**	***ni***	**mean**	**STD**	
cold sensation (°C)	26	30.08	0.93	26	29.28	1.54	0.072
warm sensation (°C)	26	34.06	0.97	26	35.01	1.79	0.016*
cold pain (°C)	25	17.80	7.23	26	20.75	6.56	0.099
hot pain (°C)	26	41.77	4.25	26	42.05	4.56	0.742
vibratory limits (μ)	26	1.58	1.33	26	1.92	1.87	0.971

	**Female**						

**Nerves**	**Median**			**Ulnar**			***p*-value**
	***ni***	**mean**	**STD**	***ni***	**mean**	**STD**	

cold sensation (°C)	35	29.65	1.41	35	29.30	1.25	0.086
warm sensation (°C)	35	33.91	0.56	35	34.00	0.48	0.667
cold pain (°C)	35	20.31	4.56	35	22.23	4.82	0.035*
hot pain (°C)	35	39.31	2.74	35	39.47	3.30	0.953
vibratory limits (μ)	35	0.98	0.39	35	0.85	0.30	0.198

*The significant *P*-value ≤ 0.05.

**TABLE 5:** Dispersion (the temperature difference between feeling pain and temperature sensation) of low (cold) and high (warm) temperatures in the median and ulnar nerves.

**Nerves**	**Median**		**Ulnar**		***p*-value**
	**mean**	**STD**	**mean**	**STD**	
Cold dispersion (°C)	10.68	6.05	7.58	5.19	0.002*
Warm dispersion (°C)	6.37	3.60	6.13	3.82	0.539

*The significant *P*-value ≤ 0.05.

The comparison of the study results in the subgroups according to side and sex was done. The right hand was characterized by a significantly lower CS threshold, and higher WS threshold in the C8 region (ulnar nerve), while the left hand had a significantly higher CP threshold in this distribution (Table 3). The differences between female and male groups in most parameters were non-significant, except for a higher WS threshold in the ulnar nerve in the male group, and a higher CP threshold in the ulnar in females (Table 4).

The correlations between parameters of sensory conduction and QST were low. The moderate negative correlation was only seen between the amplitude of sensory potentials in both nerves and separately in the ulnar nerve and WS (respectively, −0.50, and −0.66).

## Discussion

In our previous manuscript we showed significant differences between motor conduction in the median and ulnar nerves using the CVD technique. The ulnar nerve is characterized by faster conduction velocities, which indicates a greater amount of large fibers. We undertook research into sensory conduction in the median and ulnar nerve based on standard electrophysiological tests and QST to compare these two nerves in different modalities of sensation (Cruz Martínez et al., 1978; Rolke et al., 2006; Koszewicz et al., 2019).

The results of standard conduction velocity and vibratory threshold tests in both nerves were similar and did not indicate any important differences between nerves. The above tests are generally used for estimation of the function of the largest of all sensory fibers (the large myelinated axons, Aβ fibers). Sensation of vibration in the glabrous hand skin depends on the functioning of four classes of mechanoreceptors, including fast, and slow-adapting fibers, providing sensations at different frequencies. Their distribution within the skin is not equal to regional sensitivity differences over the hand. Hand skin surface responses to vibration stimuli are diversified. Perception probably depends on the contact conditions, including contact area. The spatial summation phenomenon for different frequencies of vibration also influences sensation. Therefore, our non-significant results should be interpreted with caution (Gescheider et al., 2002; Morioka and Griffin, 2005).

The estimation of temperature thresholds allows assessment of the function of smaller myelinated and unmyelinated sensory fibers–Aδ and C. WS is a type of sensation mediated by C fibers, HP by C fibers with some involvement of A-delta fibers, CS–by A-delta fibers, CP–by both C and A-delta fibers (Oh, 2003; Siao and Cros, 2003; Dimitrova et al., 2019). HP thresholds were similar in the median and ulnar nerves, and this can be interpreted as a defense against threatening nociceptive stimuli, regardless of the localization. The thresholds of CP mediated by both C and A-delta fibers are more individual, and differ in different regions of the body (Lötsch et al., 2015; Tilley and Bisset, 2017). In our study, the CP temperature was lower in the region of the median nerve’s supply, while the CS temperature was higher in this region. A higher WS temperature threshold was estimated in the regions of the ulnar nerve. Temperature perception in the region of the median nerve’s innervation is generally better with lower thresholds, but this region is not very sensitive to cold-induced pain. Similar results were achieved when we divided the study group into subgroups in terms of sides and sex.

Interesting results were achieved when we compared the differences between the temperature felt as a pain and cold or warm sensations, respectively. The difference was statistically important only for low temperatures, i.e., the temperature range between low temperatures felt as a pain and as a cold. In the median nerve the temperature range between CP and CS was much greater than that in the ulnar nerve (10.68 vs. 7.58°C). The range for high temperatures (differences between HP and WS) was the same in the median and ulnar nerves (6.37 vs. 6.13°C). High temperatures are more damaging; therefore, the HP should probably be the lowest to be safe. In the literature we only found differences for high temperature perception regarding the postural control. This requires different nerve excitability during hyperthermia; and therefore, the tibial nerve requires reduced warm stimuli compared to the median nerve, which is probably connected with different expression of slow hyperpolarization-activated cyclic nucleotide-gated (HCN) channel isoforms (Marmoy et al., 2019). Such dependencies are not known between the median and ulnar nerves.

Thermal perception depends on the intensity and duration of a thermal stimulus, and the rate with which it changes. For perception, it is important whether the stimulation is dynamic and tactile, or whether haptic exploration is used. Bilateral and symmetrical stimulation on the extremities lowers the cold and warm thresholds. Spatial acuity is generally typical for sensory systems, but the spatial localization and differentiation of thermal stimuli are generally poor. Continuous exposure to a thermal stimulus decreases responsiveness. The adaptation process is rapid–about 60 s for changes of 1°C, but it lasts much longer for more extreme temperatures, those close to the thermal pain thresholds. These (not completely explored) facts influence thermal responsiveness. Responsiveness on the hand is different than that on the forearm, but it seems to be the same within the whole hand skin surface (Green, 2009; Yang et al., 2009; Ho et al., 2011; Lötsch et al., 2015). Our results partly contradict this. The responsiveness is different mainly in relation to low temperature sensation. It can be stated that skin regions belonging to the ulnar nerve have a more “hyperalgesic” response to a cold stimulus than do areas supplied by the median nerve. This means that the CP threshold is reached at a warmer temperature (Woolf, 2011; Tilley and Bisset, 2017). “Hyperalgesic” response has been described in different pathological conditions, mainly in chronic pain, and seems to be connected with central sensitization (Arendt-Nielsen et al., 2010; Staud, 2011; Woolf, 2011). In our study, “hyperalgesic” response in the ulnar nerve was clearly seen when we calculated the dispersion in the range of low temperatures and compared hand nerves: much lower dispersion was seen in the ulnar nerve (Table 5). Statistically significant differences for cold sensation between anatomical locations have been described by different authors (Rolke et al., 2006; Green, 2009; Tilley and Bisset, 2017), but these compared proximal and distal skin areas. Dynamic stimulation interacts preferentially with central processing of cold rather than warm stimulation (Green, 2009), which could be supported by the close relationship between cold sensitivity and mechanoreception (Stevens and Green, 1978), and their common central representation (Verhagen et al., 2004). Lötsch et al. (2015) observed distinct cool and pain perceptions and hypothesized that the low temperature sensation was mediated via different afferent channels, and psychophysical responses to cold stimuli depended on complex physiological processes. Differences in cold and warm sensation have been seen in previous studies, and may be linked to a more diffuse sense of warmth than cold, greater spatial summation for warming stimuli, and generally less numerous receptors for warm temperatures (Green, 2009; Ho et al., 2011; Lötsch et al., 2015; Marmoy et al., 2019).

We are aware of the limitations of our study. Firstly, we did not use exactly the same distances (11–13 cm) between stimulating and recording electrodes during the standard sensory neurographic tests in both nerves. The lack of the distance standardization resulted from the different hand sizes, and was in accordance with the guidelines (Oh, 2003; Mallik and Weir, 2005)–stimulation site was localized at the wrist, at the second distal-most crease. Secondly, QST is a psychophysical method. It has an objective part–physical, sensory stimulation, but the response is a subjective report from the individual. The use of advanced techniques, i.e., contact-heat-evoked potentials with very advanced thermal stimulator (CHEPS) and with functional MRI capabilities, could improve the objectivity of the results (Siao and Cros, 2003; Nothnagel et al., 2017). Secondly, the study group was middle-aged and not very large. Studies should also be performed on other age groups, separately in young adults and older persons. However, the results may reflect the above-mentioned complex, central and peripheral processing of low temperature perceptions. The different compositions of small and large fibers, respectively, in the median and in the ulnar nerves, with more numerous small fibers in the median nerve, and large fibers in the ulnar nerve, might also be important in cold perception (Koszewicz et al., 2019). All these relationships probably reflect the hand function, as the motor and sensory tasks are different for the median and ulnar nerves.

In conclusion, the study confirmed the differences in the physiological sensory perception between the median and ulnar nerves, without any differences between left and right hands and sex. The median nerve is more sensitive to temperature stimulation than the ulnar nerve, but simultaneously less sensitive to pain-inducing temperature stimuli. The difference in the temperature sensation between these two nerves is better seen for cold stimulation. These findings additionally support the theory that there are physiological differences between nerves, and between the amount and distribution of large and small fibers in the human hand, which probably reflects and influences hand function. An understanding of this phenomenon seems to be important in the diagnosis of hand nerve pathology, especially entrapment syndromes (Westerman and Delaney, 1991; Melchior et al., 2007; Schmid et al., 2014). The findings could help to avoid both positive and negative false recognitions. As a result, safer and more effective surgical interventions, and individually selected, appropriate rehabilitation treatments could be easier to determine. Although our research needs to be extended, e.g., by application of CHEPS, we can state, based on the present study, and our previous findings on motor conduction in the median and ulnar nerves (Koszewicz et al., 2019), that physiological differences between these two nerves should be fully included during the examination of hand pathology (Sander et al., 1999; Nouraei et al., 2015; Wyner and Dissabandara, 2018).

## Data Availability Statement

The raw data supporting the conclusions of this article will be made available by the authors, without undue reservation.

## Ethics Statement

The studies involving human participants were reviewed and approved by Bioethics Committee Wrocław Medical University 50-367 Wrocław, ul. Pasteura 1. The patients/participants provided their written informed consent to participate in this study.

## Author Contributions

MK carried out electrophysiological investigations, data interpretation, and prepared the manuscript. MS carried out electrophysiological investigations and data acquisition. JG carried out clinical investigation and data acquisition. MW carried out statistical analysis. KS carried out acquisition of data and prepared English version of the manuscript. SB made substantial contributions to conception and designed and prepared the manuscript. All authors contributed to the article and approved the submitted version.

## Conflict of Interest

The authors declare that the research was conducted in the absence of any commercial or financial relationships that could be construed as a potential conflict of interest.
